# CANDLE SYNDROME: Orodfacial manifestations and dental implications

**DOI:** 10.1186/s13005-015-0095-4

**Published:** 2015-12-28

**Authors:** T. Roberts, L. Stephen, C. Scott, T. di Pasquale, A. Naser-eldin, M. Chetty, S. Shaik, L. Lewandowski, P. Beighton

**Affiliations:** Faculty of Dentistry, University of the Western Cape, Private Bag X08, Mitchell’s Plain, 7785 Cape Town, South Africa; Faculty of Health Sciences, University of Cape Town, Observatory, 7925 Cape Town, South Africa; Duke Global Health Institute, Pediatric Rheumatology, Global Health, Duke University Medical Center, Durham, USA

## Abstract

A South African girl with CANDLE Syndrome is reported with emphasis on the orodental features and dental management. Clinical manifestations included short stature, wasting of the soft tissue of the arms and legs, erythematous skin eruptions and a prominent abdomen due to hepatosplenomegaly. Generalized microdontia, confirmed by tooth measurement and osteopenia of her jaws, confirmed by digitalized radiography, were previously undescribed syndromic components. Intellectual impairment posed problems during dental intervention. The carious dental lesions and poor oral hygiene were treated conservatively under local anaesthetic. Prophylactic antibiotics were administered an hour before all procedures.

Due to the nature of her general condition, invasive dental procedures were minimal. Regular follow-ups were scheduled at six monthly intervals. During this period, her overall oral health status had improved markedly.

The CANDLE syndrome is a rare condition with grave complications including immunosuppression and diabetes mellitus. As with many genetic disorders, the dental manifestations are often overshadowed by other more conspicuous and complex syndromic features. Recognition of both the clinical and oral changes that occur in the CANDLE syndrome facilitates accurate diagnosis and appropriate dental management of this potentially lethal condition.

## Background

The CANDLE syndrome [MIM256040] is a rare autosomal recessive disorder in which autoinflammatory processes lead to multisystem complications. The acronym “CANDLE” pertains to Chronic Atypical Neutrophilic Dermatosis with Lipodystrophy and Elevated temperature. Other variable features include intellectual disability and short stature. Published reports are scanty and apart from macroglossia [1] no other oro-dental features have been mentioned in the literature.

The CANDLE syndrome, which is classified as a proteasome-associated autoinflammatory syndrome (PRAAS), and known as the Nakajo-Nishimura syndrome (NKJO) was delineated in 1939 by Nakajo, a medical staff member at Tohoku University in Japan. The initial syndromic features included erythematous skin lesions, clubbed fingers, periosteal thickening and cardiac insufficiency [2]. Thereafter, Nishimura et al. [3] expanded the phenotype to include hypertrophic pulmonary osteoarthropathy. Additional phenotypic features which have been reported included prominent eyes, enlarged nose and lips; elongated, broad fingers; gross wasting of the arms and legs, severe joint pains and fever that were alleviated by the use of steroids; muscle atrophy and weakness; mild mental retardation; hepatomegaly; macroglossia; short stature and calcifications of the basal ganglia are other documented syndromic manfestations [1, 4–6].

Garg et al., [7] described a syndrome with similar features to NKJO and coined the term “Joint contractures, Muscular Atrophy, Microcytic anemia, and Panniculitis-induced Lipodystrophy (JMP) syndrome”. The main difference between the NKJO and JMP syndromes is the absence of fever in JMP syndrome and the absence of seizures in NKJO [6]. Toretello et al. [8] subsequently proposed the acronym “CANDLE” and drew attention to the fact that affected persons were homozygous for an autosomal recessive gene. In a further significant development, Wang et al. 2014 [9] suggested that the CANDLE syndrome, NakajoNishimura syndrome and JMP syndrome may be clinical variants of the same genetic disorder reflecting intragenic heterogeneity in the determinant *PSMB8* gene mutations.

In this article, we have documented and reviewed the clinical manifestations in an affected girl with emphasis oro-facial features and dental implications. In this context previously undescribed abnormalities include microdontia, microstomia and diastemata have been documented. These observations will be of practical significance in dentistry.

## Case report

A South African girl born in 2001 was seen in 2013 at the age of 12 years at the St Joseph’s home for disabled children, Cape Town. She was referred to Tygerberg Dental Hospital for routine dental management.

**Fig. 1:**
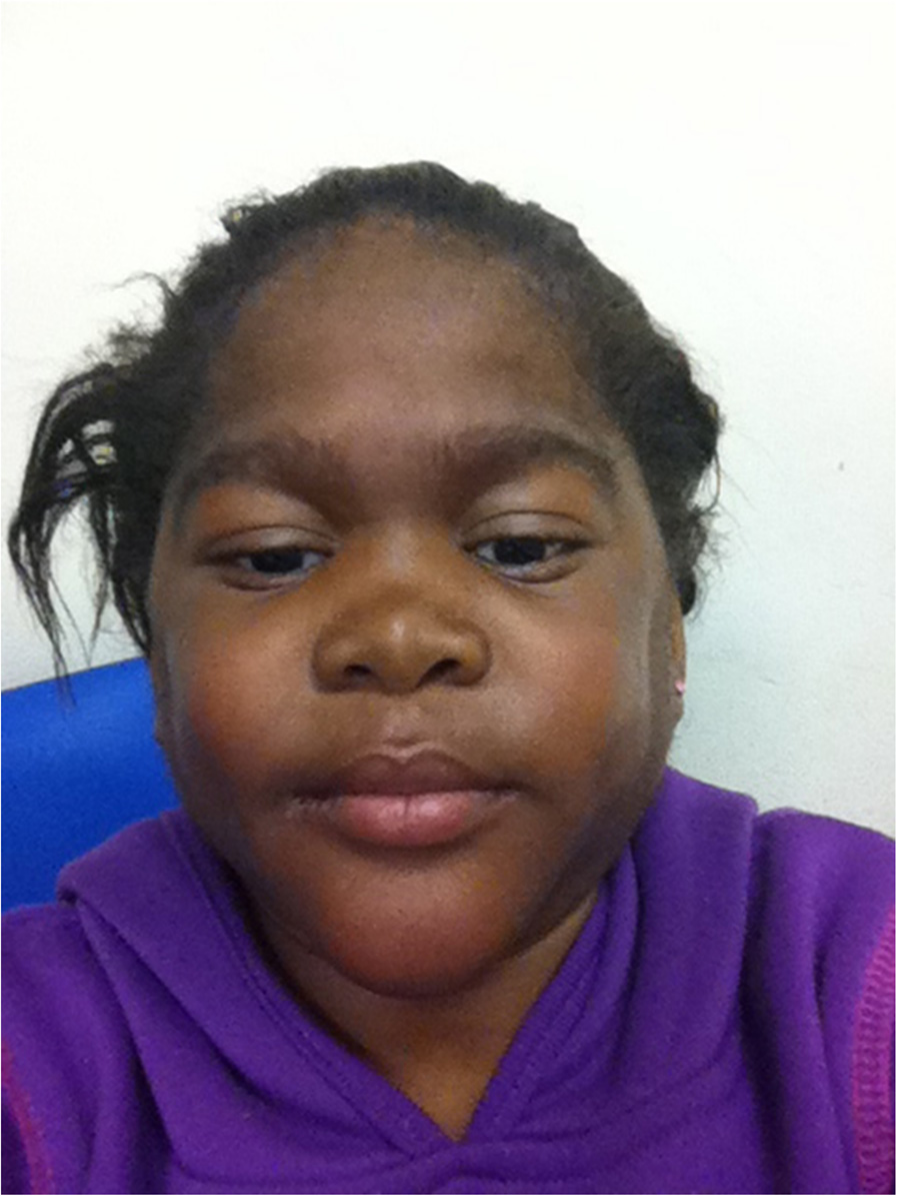
The affected girl presenting with a flattened nasal bridge

In early childhood, the affected girl received medical attention for painful progressive panniculitis, myositis and arthritis. A presumptive diagnosis of the CANDLE syndrome had previously been established on a basis of the characteristic phenotypic features of CANDLE syndrome described in medical literature, including typical facial characteristics, marked hepatosplenomegaly, fevers, lymphadenopathy, calcification of her basal ganglia on CT, and episodes of intense inflammation without infectious cause. She also suffered from delayed growth together with pronounced lipodystrophic chondritis, which resulted in a flattened nasal bridge (Fig. 1). She was severely immunocompromised as a result of immunosuppressant drugs. She also had type II diabetes mellitus, gastric reflux and had a history of tuberculosis during infancy.

### Extra-oral examination

At the age of 12 years she had short stature, with broad, thick fingers, wasting of the soft tissue of her arms and legs and an enlarged abdomen due to hepatosplenomegaly. Her facial features were coarse. Diffuse erythematous skin plaques were evident on her arms and limbs. There was no previous history of dental problems but marked oedema was noted around the perioral and nasal area. The mandibular symphyses were prominent and microstomia was present. There was no evidence of jaundice, anemia, cyanosis or clubbing. Cervical lymph nodes were palpable on the right side of the neck.

### Intra-oral examination

**Fig. 2:**
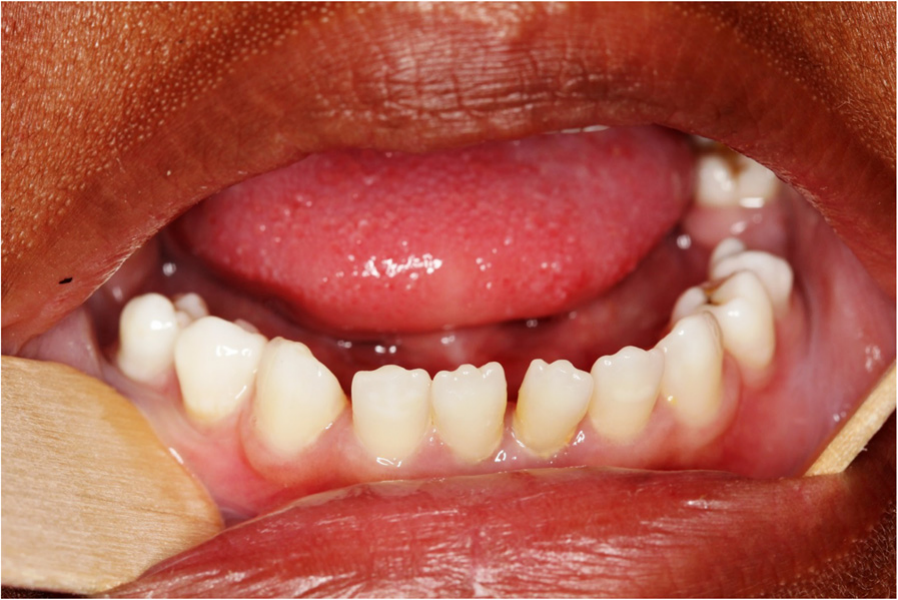
Generalized microdontia, spacing of teeth and mammelons affecting the permanent dentition

The oral soft tissues appeared unremarkable but bleeding occurred when probing the gingival margins corresponding to teeth 21, 37 and 47. Generalized spacing of her teeth and a Class III malocclusion (incisal classification) were evident. All teeth showed microdontia and mamelons were present on the incisal surfaces. (Fig. 2). Both first mandibular molars were absent possibly as a consequence of previous extractions.

## Special investigations

### Laboratory studies

Numerous investigations for autoimmune and infectious diseases had been undertaken. These included Anti-Nuclear antibodies *ANA* (3 consecutive tests), anti-DNA antibodies and other auto-antibodies. She had a normal white blood cell count, mild microcytic anemia, and mild elevation of platelet levels. These investigations all yielded negative results. Inflammatory markers and serum triglycerides were elevated. At times of flare, transient elevation of muscle enzymes CK, AST and ALT had occurred. Her uric acid levels were normal. She had been investigated several times for HIV with negative results, given that these infections are highly prevalent in South Africa. Investigations for infectious disorders included tests for bacterial, viral, parasitic, and fungal infections; all were negative. The syphilis RPR test was non-reactive. Urine studies yielded normal results. Abdominal ultrasonic studies confirmed the presence of hepatosplenomegaly. A CT scan of her brain was undertaken shortly after a seizure, revealing calcifications of her basal ganglia, but no other signs of a mass lesion or inflammation. Histopathological investigations of multiple tissues showed diffuse neutrophilic infiltrates at multiple sites including muscle, liver and skin.

She had significantly increased levels of Interferon gamma (IFN-y).

### Dental radiography

**Fig. 3:**
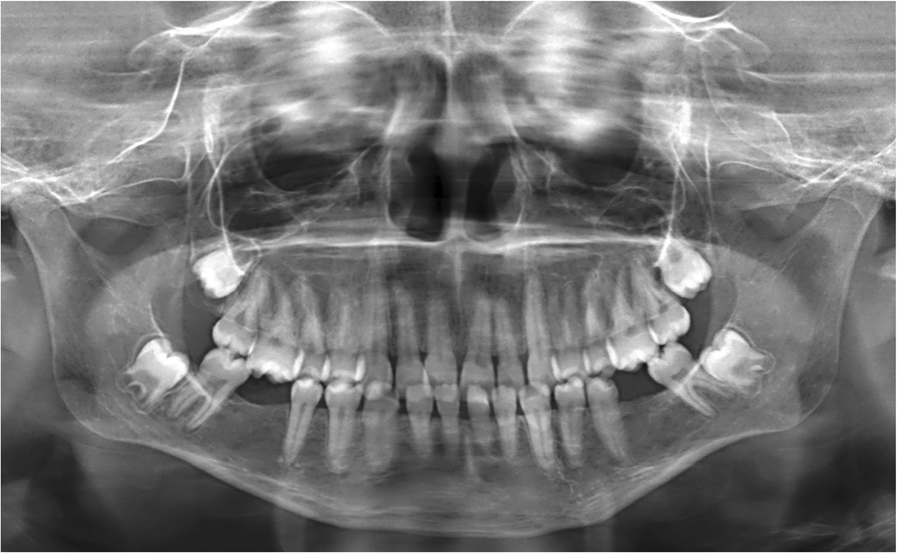
A panorex image of the jaws showing generalized microdontia

A panoramic view (64 kV, 112 mAs) confirmed missing teeth 36 and 46, and revealed caries on teeth 15, 25 and 21. There was generalized spacing of mandibular dentition and an overerupted first maxillary right molar (Fig. 3). The radiologist also noted generalized osteopenia of the mandible. Further investigations were undertaken to confirm the presence of osseous changes and to establish the magnitude of possible osteopenia\osteoporosis. According the WHO, osteopenia is defined as “bone density measurements (T score) between 1 and 2.5 standard deviations below the young adult mean” [10]. To avoid exposing the young patient to further radiation exposure and as the panorex radiograph was already available, the bone mineral density (BMD) was determined by digitalizing and analyzing the dental images. The BMD of the mandible correlates favourably with that found in the lumbar spine and neck of the femur which are the conventional sites of BMD measurements [11, 12]. Both linear and densitometric measurements were obtained using an analytical software package (J Image). The Panoramic Mandibular Index (PMI), Klemmeti Index and Mandibular Cortical Width (MCW) were measured as described by Mansour et al. [13]. The mean pixel intensity that is the amount of the radiolucency or radioopacity of a region on the radiograph on a gray scale from zero (complete radioopacity) to the highest value (complete radiolucency) was determined.

The Klemmeti Index measures the morphology of the mandibular cortex and is conventionally categorized as C1 (normal), C2 (osteopenic), or C3 (osteoporotic).

Since standard values for bone mineral density in children are available, the results were compared to two females of similar age and ethnicity using the same exposure values (Table 1). All results indicate that the BMD of the affected girl was much less than the two girls of same age, gender and ethnicity.

**Table 1:** Bone mineral density measurements of the girl with Candle syndrome compared with two unaffected females of same age, ethnicity and gender

Densitometric analysis	Affected girl	Control 1	Control 2
Mean PI	78.65	94.2	96.3
Morphometric analysis			
Mandibular cortical width	0.66 mm (mean)	2.08 mm (mean)	2.81 mm (mean)
Klemmeti index	C 3	C 1	C2
Panoramic mandibular Index	Not applicable		

**Fig. 4:**
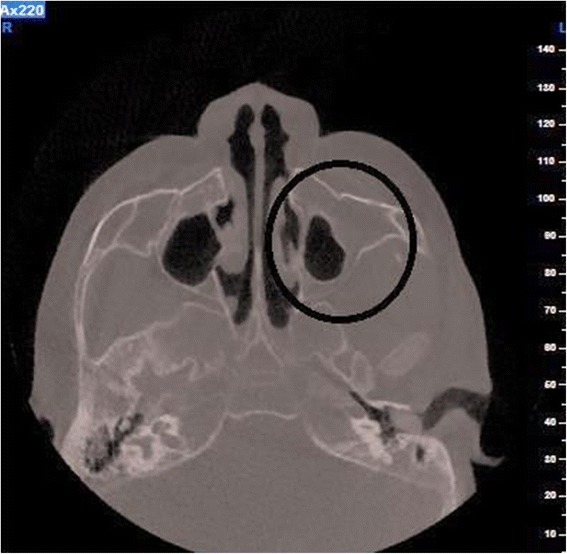
Cone Beam Tomography showing hypoplasia of both maxillary sinuses

Cone beam tomography (CBCT; 110 kV, 3.46 mAs) was used to ascertain whether additional complications were present. The results of this investigation revealed that the frontal and sphenoidal sinuses were absent. Both maxillary sinuses were hypoplastic, the left side to a greater degree (Fig. 4). Bilateral opacification of the external auditory canals was noted. There was bilateral widening of the diploeic space in the lesser wing of the sphenoid bone and bilateral widening of the diploeic space of the maxilla, the left side being more prominent than the right.

### Tooth size analysis

Alginate impressions of both the maxillary and mandibular arches were made and the impressions were immediately poured into a laboratory dental stone. All measurements were taken directly from the unsoaped plaster study models. The teeth measured included the maxillary and mandibular permanent central and lateral incisors, the maxillary and mandibular permanent canines, first and second premolars and the maxillary first molars.

A sliding manual caliper was used to measure the mesiodistal tooth width according to the guidelines defined by Hunter and Priest [14].

The results of the measurements were compared with unpublished data from a report of research conducted in South Africa (unpublished data di Pasquale 2012). All the affected girl’s teeth were smaller than the mean of the sample (Table 2). There was a total reduction in the size of 7.47mm in each quadrant in the maxilla and 8.14 mm in each quadrant in the mandible. This is s a clinically important difference and could be considered to be diagnostic of microdontia.

**Table 2:** Comparison of normal tooth size in black South African females with patient’s tooth size

Unpublished data				Patient's measurements				
Tooth	Mean	Std. deviation	Minimum	Maximum	Right	Left	Mean	Difference
Upper 6	10.50	0.41	9.65	11.15	9.50	10.20	9.85	0.65
Upper 5	6.97	0.38	6.21	7.67	5.50	5.10	5.30	1.67
Upper 4	7.55	0.43	6.70	8.54	6.00	6.50	6.25	1.30
Upper 3	7.88	0.41	6.83	8.74	7.00	7.00	7.00	0.88
Upper 2	7.28	0.53	5.85	8.01	6.20	6.10	6.15	1.13
Upper 1	9.04	0.49	7.80	10.20	6.80	7.60	7.20	1.84
Lower 6	11.47	0.56	10.36	12.77				
Lower 5	7.59	0.44	6.71	8.52	5.00	5.60	5.30	2.29
Lower 4	7.64	0.43	6.85	8.59	6.00	5.90	5.95	1.69
Lower 3	7.10	0.31	6.54	7.86	6.10	6.10	6.10	1.00
Lower 2	6.13	0.33	5.57	6.86	4.90	4.10	4.50	1.63
Lower 1	5.47	0.31	4.72	6.35	3.90	4.00	3.95	1.52

## Dental management

The carious lesions and poor oral hygiene were treated conservatively under local anaesthetic. Prophylactic antibiotics were administered an hour before all interventions and due to the nature of her general condition, invasive procedures were .avoided where possible. Regular follow-ups were scheduled at six monthly intervals. During this period, her overall oral health status had improved markedly.

### Consent

Written informed consent was obtained from the patient’s legal guardian for publication of this case report and any accompanying special investigations and images. A copy of the written consent is available for review by the Editor-in-Chief of this journal.

## Discussion

Our approach to the documentation and discussion of the orofacial and dental manifestations of the CANDLE syndrome was constrained by the rarity of the disorder as only approximately 30 cases have been reported in the literature. The strength of our approach is the combination of expertise of the authors, involving different scientific, dental and clinical disciplines.

### Pathogenesis

The CANDLE syndrome is caused by homozygosity for mutations in the Proteasome (Prosome, Macropain) Subunit, Beta Type, 8 (*PSMB8*) gene that encodes for proteasomes that are responsible for the physiological degradation of proteins. Mutations of *PSMB8* result in an accumulation of modified and oxidated proteins in cells and tissues, leading to an increase of cellular stress and increased apotosis occurring in muscle and fat [1, 15–17]. Recent developments indicate that not all individuals affected by the CANDLE syndrome have *PSMB8* mutations [18]. Brehm et al. 2015 identified 8 mutations in 4 proteasome genes, *PSMA3*, *PSMB4, PSMB9*, and proteasome maturation protein (*POMP*), that have not previously been related to the disease. These mutations affect transcription, protein expression, protein folding, proteasome assembly, and, eventually, proteasome activity [19].

### Microdontia

Microdontia has previously not been reported in CANDLE syndrome. This developmental abnormality can involve either the primary or permanent dentition and as a component of a few genetic syndromes (Table 3). Microdontia can also occur in non- genetic conditions notably as a complication of radiation or chemotherapeutic treatment [20].

**Table 3:** Microdontia in genetic syndromes

Syndromic conditions	Reference
Gorlin-Chaudhry-Moss syndrome	[28]
William syndrome	[29]
Turner syndrome	[30]
Rothmund-Thomson syndrome	[31, 32]
Seckel syndrome	[33, 34]
Spondyloepiphyseal dysplasia	[35, 36]
Kenny-Caffey Syndrome.	[37]
Coffin-Lowry syndrome	[38]
Microcephalic osteodysplastic primordial dwarfism	[39, 40]

Shafer et al. [21] classified microdontia into three categories viz:

- True generalized microdontia in which all the teeth are smaller than normal is rare.
- Relative generalized microdontia occurs when teeth are normal in size, but appear to be smaller than normal. For example, if the jaws are large and teeth are normal.
- Microdontia involving a single tooth.

#### The genetic basis of microdontia

Over 300 genes are implicated in tooth development [22]. Many of these genes regulate ectodermal-mesenchymal interactions in a programmed sequence. In turn, these processes control the shape, number and sizes of teeth. Similar ectodermal-mesenchymal interactions occur throughout the developing fetus and in many instances, involve the same genes. For these reasons, the occurrence of dental anomalies in genetic syndromes could be an indicator of common developmental factors in both dental and other tissues [23].

The relationship between growth and tooth size indicates that repeated ectodermal– mesenchymal interactions occur during the initiation and morphogenesis phases of tooth development. Although epigenetic influences affect the position of tooth forming tissue within the jaw, scheduling of the communicating signals explains differences in tooth size. In this context, no particular gene has been implicated as the primary cause of microdontia.

Although a decrease in bone density is commonly associated with the long-term use of corticosteroids, there is no documented evidence to suggest that steroids influence odontogenesis.

### Microstomia

Microstomia refers to a decrease in the size of the opening of the mouth. Although there are no standardized criteria to measure the extent of mouth opening, microstomia affects both function and aesthetics [24]. Microstomia can result in to difficulty in swallowing, speech impairment, deficient oral hygiene and dental caries. In the affected girl, small dimensions of the oral orifice compromised dental management scaling and polishing.

### Diastemata

The term refers to increased spacing between teeth and is often caused by loss of interproximal contact between teeth. The most common site of diastemata is in the anterior maxilla between the cuspid teeth [25]. Generalized increase in interdental spacing occurs when there is a disproportionate relationship between the size of the teeth and that of the jaw [26]. Interproximal tooth wear may be a contributing factor. The generalized spacing of the affected girl’s teeth was probably the result of the microdontia.

### Hypoplastic air sinuses

Hypoplasia of the cranial sinuses was evident on CBCT investigation of the affected girl. Sinuses serve several functions: they decrease the weight of the anterior aspect of the skull, increase the resonance of the voice, have a protective role by dampening pressure (e.g. due to trauma to the face), increase the rigidity of the facial bones and serve to protect structures such as the eyes. They also filter and humidify the air during respiration [27].

### Osteopenia

The results of the both densometric and linear measurements suggest that osteopenia was present in the affected girl. It is uncertain however, whether the osteopenia resulted from the long-term use of systemic corticosteroid or whether it is a previously unreported syndromic component. Periodontal disease, decreased alveolar bone density and edentulism are frequent in persons affected by osteoporosis. Fractures of the jaw can result in impaired function, affecting the individual’s quality of life.

### Dental management considerations in the CANDLE syndrome

The presence of severe immunosuppression that was compounded by diabetes mellitus in the affected girl was a matter of concern. In these circumstances, a multi-disciplinary approach was necessary for the provision of dental management. Factors that warranted consideration when planning her dental treatment included diet, blood glucose levels, reduced leukocyte function Decreased integrity of the blood vessels, which is a common complication of diabetes mellitus was also relevant. In addition, immunosuppressive drugs could result in bone marrow suppression and decrease the production of platelets and leukocytes as well as induce osteopenic changes that could predispose to jaw fractures. Together, these factors increase the risk of developing infection, delayed wound healing and prolonged bleeding times. In these circumstances, the dental management of the affected girl was by conventional procedures with an increased awareness of the risks of possible complications.

## Conclusion

The CANDLE syndrome is a rare condition with grave complications including immunosuppression and diabetes mellitus. As with many genetic disorders, the dental manifestations are often overshadowed by other more conspicuous and complex syndromic features. Recognition of both the clinical and oral changes that occur in the CANDLE syndrome facilitates accurate diagnosis and appropriate management of this potentially lethal condition.

All investigations were undertaken with full ethical approval in accordance with the Declaration of Helsinki as updated in the version promulgated in June 2013 and the Singapore Statement on Research Integrity. Ethics approval was received from the University of Cape Town Faculty of Health Sciences Institutional Ethics Committee (no. 203/2013).
